# Supplementary material for: Trajectories of disability and influence of contextual factors among adults aging with HIV: Insights from a community-based longitudinal study in Toronto, Canada
Source: PLoS One. 2025 Dec 9;20(12):e0309575. doi: 10.1371/journal.pone.0309575 (PMC12688091; doi:10.1371/journal.pone.0309575)
Supplement: S1 Fig — (PDF) [file pone.0309575.s001.pdf]

Supplementary Figure 1 – Trajectories of disability and influence of contextual factors among adults aging with HIV: insights from a community-based longitudinal study in Toronto, Canada

S1 Fig. Overview of study design and measurement timepoints during the baseline monitoring phase of the community-based exercise (CBE) intervention study

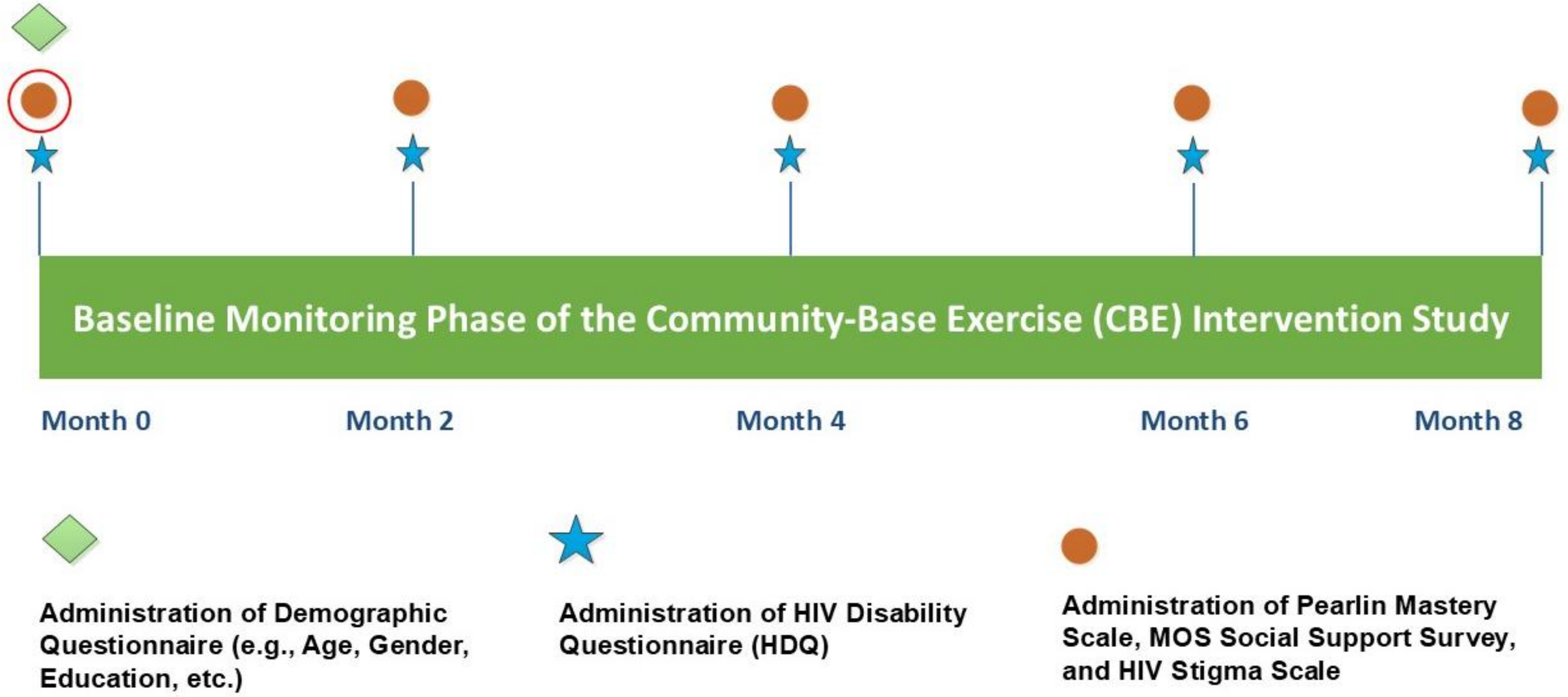

Note: The present study draws on data from the 8-month baseline monitoring phase of the CBE intervention study, during which disability was assessed bi-monthly (5 timepoints; https://doi.org/10.1136/bmjopen-2016-013618). All questionnaires were administered electronically. The demographic questionnaire was administered once at baseline (Month 0). Self-mastery, social support, and HIV stigma questionnaires were administered across five measurement occasions; however, we used baseline values in this study to examine how these contextual factors were associated with subsequent disability trajectories (depicted as the first dot with an outer circle in S1 Fig.).
