# Supplementary material for: Trajectories of disability and influence of contextual factors among adults aging with HIV: Insights from a community-based longitudinal study in Toronto, Canada
Source: PLoS One. 2025 Dec 9;20(12):e0309575. doi: 10.1371/journal.pone.0309575 (PMC12688091; doi:10.1371/journal.pone.0309575)
Supplement: S2 Fig — (PDF) [file pone.0309575.s002.pdf]

Supplementary Figure 2 – Trajectories of disability and influence of contextual factors among adults aging with HIV: insights from a community-based longitudinal study in Toronto, Canada

S2 Fig. The interconnections between trajectories across the six disability dimensions

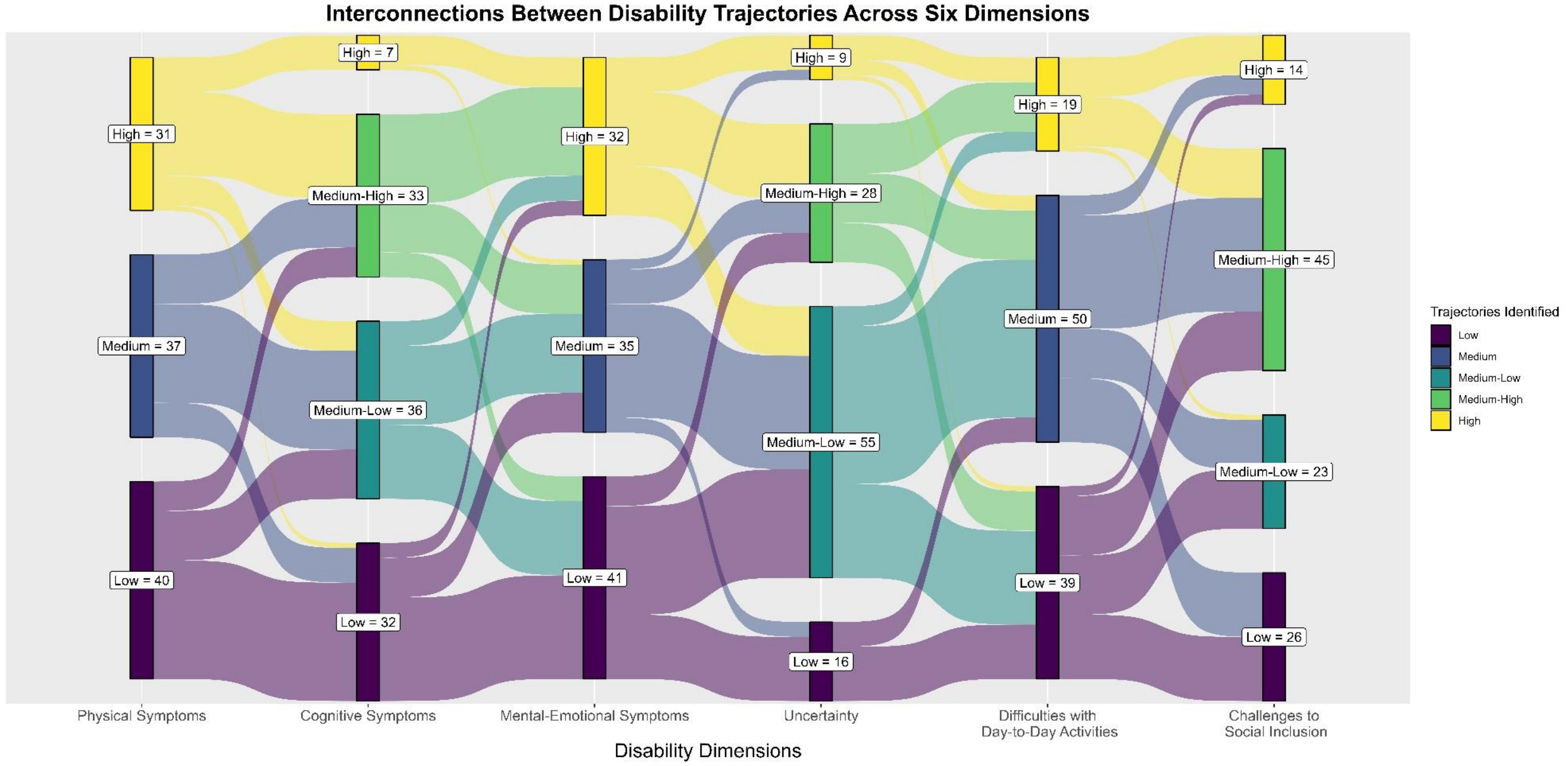

Date Last Revised: November 11, 2025
