# Supplementary material for: Trajectories of disability and influence of contextual factors among adults aging with HIV: Insights from a community-based longitudinal study in Toronto, Canada
Source: PLoS One. 2025 Dec 9;20(12):e0309575. doi: 10.1371/journal.pone.0309575 (PMC12688091; doi:10.1371/journal.pone.0309575)
Supplement: S1 Table — (PDF) [file pone.0309575.s003.pdf]

S1 Table. Characteristics of participants who initiated and remained at the end of baseline monitoring phase (n = 83)

Participant characteristics	Mean ± SD / n (%)
Age (years)	51.5 ± 10.8
Gender (n)	
Men	76 (92)
Women	7 (8)
Race/Ethnicity	
White	54 (65)
Black or African	5 (6)
Hispanic or Latino	4 (5)
Number of years since HIV diagnosis	17.9 ± 10.2
Self-reported undetectable HIV viral load (<50 copies/mL)	72 (87)
Current use of antiretroviral medications	83 (100)
Education (some university+)	41 (49)
Living alone (n)	53 (64)
Number of comorbidities in addition to living with HIV (count)	5.0 ± 4.2
Three most common comorbidities	
Mental health condition (e.g., depression, anxiety)	36 (43)
Joint pain (arthritis)	35 (42)
Bone and joint disorder (e.g., osteonecrosis, osteopenia)	31 (37)
Pearlin Mastery Scale score (range: 7–28)	19.9 ± 4.0
HIV Stigma Scale total score (range: 40–160) †	93.9 ± 23.7
Personalized stigma subscale score (range: 18–72)	40.4 ± 11.6
Disclosure concerns subscale score (range: 10–40)	25.7 ± 7.0
Negative self-image subscale score (range: 13–52)	27.8 ± 8.5
Public attitudes subscale score (range: 20–80)	46.7 ± 12.6
MOS Social Support Survey total score (range: 0–100) ‡	56.5 ± 23.8
Emotional and information support raw score (range: 8–40)	26.9 ± 7.4
Tangible support raw score (range: 4–20)	12.4 ± 5.6
Affectionate support raw score (range: 3–15)	9.5 ± 4.0
Positive social interaction raw score (range: 4–20)	13.1 ± 4.5

Notes: SD = Standard deviation. †Sixteen items on the HIV Stigma Scale belong to more than one subscale, which reflects the intercorrelations between different subscales. ‡ Raw scores of the MOS Social Support Survey were summed across the four subdomains and transformed into a 100-point scale. Greater Pearlin Mastery Scale scores, HIV Stigma Scale scores, and MOS Social Support Survey scores reflect higher levels of self-mastery, HIV-related stigma, and perceived social support, respectively
