# Supplementary material for: Trajectories of disability and influence of contextual factors among adults aging with HIV: Insights from a community-based longitudinal study in Toronto, Canada
Source: PLoS One. 2025 Dec 9;20(12):e0309575. doi: 10.1371/journal.pone.0309575 (PMC12688091; doi:10.1371/journal.pone.0309575)
Supplement: S2 Table — (PDF) [file pone.0309575.s004.pdf]

S2 Table. Trajectory coefficients for physical, mental-emotional symptoms, and day-to-day activity difficulties

	Low trajectory		Medium trajectory		High trajectory	
	Physical symptoms					
Model parameters	*b*	SE	*b*	SE	*b*	SE
Intercept	18.01***	1.45	30.48***	1.98	42.76***	1.84
Linear slope	-0.06	0.03	-0.01	0.03	-0.02	0.03
Quadratic slope	0.00	0.00	0.00	0.00	0.00	0.00
	Mental-emotional symptoms					
Model parameters	*b*	SE	*b*	SE	*b*	SE
Intercept	19.42***	2.13	35.31***	4.02	57.85***	2.93
Linear slope	-0.08*	0.01	0.02	0.05	-0.02	0.05
Quadratic slope	0.00*	0.00	-0.00	0.00	-0.00	0.00
	Difficulties with day-to-day activities					
Model parameters	*b*	SE	*b*	SE	*b*	SE
Intercept	4.98***	1.37	14.53***	1.37	35.64***	2.21
Linear slope	-0.04	0.03	0.02	0.02	-0.03	0.04
Quadratic slope	0.00	0.00	-0.00	0.00	0.00	0.00

Notes: * $p<.05$. ** $p<.01$. *** $p<.001$
