# Supplementary material for: Trajectories of disability and influence of contextual factors among adults aging with HIV: Insights from a community-based longitudinal study in Toronto, Canada
Source: PLoS One. 2025 Dec 9;20(12):e0309575. doi: 10.1371/journal.pone.0309575 (PMC12688091; doi:10.1371/journal.pone.0309575)
Supplement: S3 Table — (PDF) [file pone.0309575.s005.pdf]

S3 Table. Trajectory coefficients for cognitive symptoms, uncertainty, and challenges to social inclusion

	Low trajectory		Medium-low trajectory		Medium-high trajectory		High trajectory	
	Cognitive symptoms							
Model parameters	*b*	SE	*b*	SE	*b*	SE	*b*	SE
Intercept	7.86***	1.94	18.24***	1.92	33.65***	2.01	74.21***	5.00
Linear slope	-0.06	0.03	-0.04	0.03	0.03	0.04	-0.30**	0.10
Quadratic slope	0.00	0.00	0.00	0.00	-0.00	0.00	0.00*	0.00
	Uncertainty							
Model parameters	*b*	SE	*b*	SE	*b*	SE	*b*	SE
Intercept	7.50*	3.03	35.53***	1.64	50.26***	2.75	81.19***	5.06
Linear slope	-0.03	0.05	-0.09**	0.03	0.02	0.04	-0.15	0.09
Quadratic slope	0.00	0.00	0.00**	0.00	-0.00	0.00	0.00	0.00
	Challenges to social inclusion							
Model parameters	*b*	SE	*b*	SE	*b*	SE	*b*	SE
Intercept	13.46***	0.03	30.03***	1.43	40.25***	1.11	48.98***	1.75
Linear slope	-0.12***	0.03	-0.09**	0.03	-0.04	0.02	0.02	0.03
Quadratic slope	0.00***	0.00	0.00*	0.00	0.00	0.00	-0.00	0.00

Notes: * $p<.05$. ** $p<.01$. *** $p<.001$
