# Supplementary material for: Trajectories of disability and influence of contextual factors among adults aging with HIV: Insights from a community-based longitudinal study in Toronto, Canada
Source: PLoS One. 2025 Dec 9;20(12):e0309575. doi: 10.1371/journal.pone.0309575 (PMC12688091; doi:10.1371/journal.pone.0309575)
Supplement: S4 Table — (PDF) [file pone.0309575.s006.pdf]

S4 Table. Average posterior probability of group assignment

Disability dimensions	Final number of trajectories	Posterior probability of group assignment			
		Group 1	Group 2	Group 3	Group 4
Physical symptoms	3	0.867	0.907	0.901	
Cognitive symptoms	4	0.968	0.847	0.945	0.920
Mental-emotional symptoms	3	0.939	0.837	0.862	
Uncertainty	4	0.983	0.921	0.885	0.936
Difficulties with day-to-day activities	3	0.897	0.906	0.966	
Challenges to social inclusion	4	0.943	0.945	0.905	0.964
