# Supplementary material for: Trajectories of disability and influence of contextual factors among adults aging with HIV: Insights from a community-based longitudinal study in Toronto, Canada
Source: PLoS One. 2025 Dec 9;20(12):e0309575. doi: 10.1371/journal.pone.0309575 (PMC12688091; doi:10.1371/journal.pone.0309575)
Supplement: Attachment — Submitted filename: Reviewers feedback .docx [file pone.0309575.s008.docx]

**Overall Impression**

This is a strong and good manuscript that effectively communicates the purpose, methods, key findings, and implications of the research. It is well-suited for a scientific audience and clearly demonstrates the value of the study. The methodology is sophisticated and appropriate for the research questions.

By incorporating more descriptive language for the trajectories and using stronger, more causal language to describe the associations, the authors can elevate an already excellent abstract to be even more compelling and informative.

**Strengths**

1. **Clarity and Conciseness:** The abstract is well-organized following the standard IMRaD (Introduction, Methods, Results, Conclusion) structure. Each section is clear and to the point, making it easy for the reader to follow the study's narrative.
2. **Strong and Relevant Research Question:** The focus on the *trajectories* of disability, rather than just its prevalence at a single point in time, is a significant strength. This longitudinal approach is crucial for understanding the lived experience of a chronic condition like HIV in an aging population.
3. **Sophisticated and Appropriate Methodology:**
   - The use of **Latent Class Growth Analysis (LCGA)** is a major strength. It moves beyond simply looking at average group changes and acknowledges that the population is not homogeneous, identifying distinct subgroups ("trajectories") with different experiences of disability over time.
   - The inclusion of both **intrinsic and extrinsic contextual factors** provides a holistic view of what influences disability, aligning with contemporary biopsychosocial models of health.
   - The use of a validated, HIV-specific instrument (the SF-HDQ) strengthens the validity of the disability measurements.
4. **Important and Actionable Findings:**
   - The key finding of **heterogeneity** (3-4 distinct trajectories per dimension) is powerful. It counters a potential assumption that all people aging with HIV have a uniformly bad (or good) experience and highlights the need for personalized care.
   - Identifying specific, modifiable factors like **self-mastery, social support, and stigma** as key influencers provides clear targets for clinical and community-based interventions. This moves from description to potential action.
5. **Clear Conclusion:** The conclusion directly links the findings back to clinical/practical implications, stating that understanding these contextual factors can help "inform interventions and supports."

**Weaknesses & Areas for Improvement**

While the abstract is strong, there are areas where clarity could be enhanced or details added to strengthen its impact.

1. **Lack of Specificity in Results:**
   - The abstract states that trajectories were found but doesn't describe their *nature*. For example, what does "high-declining" mean in the social inclusion dimension? Did it improve or worsen? A brief descriptor for the key trajectories (e.g., "a 'high-stable' trajectory," "a 'low-improving' trajectory") would add significant meaning.
   - The results for the multinomial regression are vague. Stating that factors "were associated with" trajectories is weak. A more impactful statement would specify the direction and, if space allows, the strongest predictors (e.g., "Higher stigma was the strongest predictor of belonging to the 'high-severity' physical disability trajectory").
2. **Sample Description and Generalizability:**
   - The sample is overwhelmingly male (89%). While this may reflect the epidemiology of the aging HIV population in the study context, it is a limitation that should be briefly acknowledged, as the findings may not be generalizable to women aging with HIV.
   - The mean age of 50.6 is relatively young for "aging" in many gerontological studies. A brief justification for this age cutoff (e.g., the premature aging observed in HIV) would be helpful for a broader audience.
3. **Methodological Detail (Minor):**
   - The number of participants (n=108) is modest for a longitudinal study with 5 time points and a complex statistical model (LCGA). While not necessarily a flaw, it's a contextual factor that a critical reader would note. The authors are likely confident in their model fit, but it's an inherent limitation of the study design.
   - It is unclear if the contextual factors were measured only at baseline (which is implied) or also longitudinally. Using only baseline measures is common, but it limits the ability to model how *changes* in social support or stigma, for example, might influence disability trajectories.

**Specific Feedback and Suggestions for Revision**

Here is how the results and conclusion could be slightly revised for greater impact:

**Original Results Snippet:**

"We identified three disability trajectories: low, medium, and high disability severity... Factors such as higher self-mastery and social support were associated with lower disability trajectories, whereas greater number of comorbidities and stigma were associated with more severe disability trajectories..."

."

**Original Conclusion:**

"Information on contextual factors may be helpful for informing interventions..."

**Suggested Strengthened Conclusion:**

"The heterogeneity in disability trajectories underscores the need for personalized care. **Targeting modifiable factors like stigma (e.g., through anti-stigma campaigns) and bolstering self-mastery and social support represent promising avenues for interventions** designed to mitigate disability among adults aging with HIV."
