# Supplementary material for: Trajectories of disability and influence of contextual factors among adults aging with HIV: Insights from a community-based longitudinal study in Toronto, Canada
Source: PLoS One. 2025 Dec 9;20(12):e0309575. doi: 10.1371/journal.pone.0309575 (PMC12688091; doi:10.1371/journal.pone.0309575)
Supplement: Attachment — Submitted filename: PONE-D-24-33533_R2_ResponseLetter_SUBMITTED.docx [file pone.0309575.s009.docx]

Dear Dr. Eneh,

Thank you for the opportunity to revise and resubmit our manuscript entitled “Trajectories of disability and influence of contextual factors among adults aging with HIV: insights from a community-based longitudinal study in Toronto, Canada” (PONE-D-24-33533R1) to PLOS ONE. My co-authors and I sincerely thank the editor and reviewers for their thoughtful and constructive comments on our manuscript.

We uploaded a response letter with point-by-point responses to comments made by the editor and reviewers. Our revisions are marked in red within the manuscript. Thank you for your consideration.

Sincerely,

Kelly O’Brien, PhD, PT
Professor, Department of Physical Therapy, University of Toronto
[kelly.obrien@utoronto.ca](mailto:kelly.obrien@utoronto.ca)

Review Comments to the Author:

**Editor**

*1) I have evaluated the original and revised manuscript(s) and can confirm that your methodology is sound. However, I notice that the current manuscript is closely linked to O'Brien et al. (2016) https://doi.org/10.1136/bmjopen-2016-013618. To further strengthen your methods section, I recommend including a flow chart that illustrates the data source, showing how data were collected, the five time points, and the measures used. This will make the methodology clearer and more accessible to readers.*

**RESPONSE-1**: Thank you for the positive feedback and suggestion. We developed a flow chart illustrating the study design, data sources, five measurement timepoints, and corresponding measures from the community-based exercise (CBE) study. We added this figure to the revised manuscript as Supplementary Figure S1 (Page 7, lines 134-135).

============================

**Reviewer #1**

*1) All comments via peer review have been fully considered and addressed by the study authors. The paper is suitable for publication and will add value to the literature base in the field of HIV, disability and rehabilitation.*

**RESPONSE-1**: We thank the reviewer for providing these positive comments. We appreciate your valuable suggestions and recognition of our investigation.

============================

**Reviewer #3**

*This is a strong and good manuscript that effectively communicates the purpose, methods, key findings, and implications of the research. It is well-suited for a scientific audience and clearly demonstrates the value of the study. The methodology is sophisticated and appropriate for the research questions. By incorporating more descriptive language for the trajectories and using stronger, more causal language to describe the associations, the authors can elevate an already excellent abstract to be even more compelling and informative.*

*1) The abstract states that trajectories were found but doesn't describe their nature. For example, what does "high-declining" mean in the social inclusion dimension? Did it improve or worsen? A brief descriptor for the key trajectories (e.g., "a 'high-stable' trajectory," "a 'low-improving' trajectory") would add significant meaning.*

**RESPONSE-1**: Thank you for the thoughtful observation and opportunity to clarify. The identified disability trajectories primarily differed in their baseline levels (intercepts) and remained relatively stable over time. To improve clarity and ensure consistent terminology across dimensions, we revised the label “high-declining” to “high” in the cognitive symptom dimension during the previous round of review (R1). This revision is reflected in the current manuscript. We discussed the implications of these findings in the Discussion section (Page 24, lines 379-400).

*2) The results for the multinomial regression are vague. Stating that factors "were associated with" trajectories is weak. A more impactful statement would specify the direction and, if space allows, the strongest predictors (e.g., "Higher stigma was the strongest predictor of belonging to the 'high-severity' physical disability trajectory").*

**RESPONSE-2**: Thank you for the insightful comment. In our analyses, we used information on contextual factors measured at baseline. Thus, while associations can be identified, we were unable to strictly determine causal direction, which we acknowledged as a limitation (Page 27, lines 454-456). Because each contextual factor was measured on different scales (e.g., continuous for age and categorical for gender), direct comparison of effect magnitudes is challenging. Hence, we focused on describing the direction and statistical significance of each association when interpreting the findings.

*3) The sample is overwhelmingly male (89%). While this may reflect the epidemiology of the aging HIV population in the study context, it is a limitation that should be briefly acknowledged, as the findings may not be generalizable to women aging with HIV.*

**RESPONSE-3**: Following the suggestion, we revised the Limitations section to acknowledge the limited gender representation in our sample. The revised sentence now reads: “Given the characteristics of the participants in this study, findings may not be transferable to individuals of different genders, racial/ethnic backgrounds, and those living in rural areas or with diverse sociodemographic characteristics.” (Page 27, lines 451-454)

*4) The mean age of 50.6 is relatively young for "aging" in many gerontological studies. A brief justification for this age cutoff (e.g., the premature aging observed in HIV) would be helpful for a broader audience.*

**RESPONSE-4**: Thank you for this thoughtful comment. We did not apply an age cutoff to define aging and instead conceptualized it as a process that unfolds over time while living with a complex chronic condition such as HIV. In our sample, participants had lived with HIV for an average of 17.5±9.9 years, reflecting prolonged exposure to the health, functional, and social challenges associated with aging with HIV over the lifespan. The mean age of 50.6 years in our study is similar to the mean age reported in a population-based study of people living with HIV in Ontario, Canada (mean = 51.3 years; Coelho et al., 2025). In response to your suggestion, we included the corresponding sentences in the revised manuscript (Page 23, lines 356-359).

*5) The number of participants (n=108) is modest for a longitudinal study with 5 time points and a complex statistical model (LCGA). While not necessarily a flaw, it's a contextual factor that a critical reader would note. The authors are likely confident in their model fit, but it's an inherent limitation of the study design.*

**RESPONSE-5**: We clarified sample size as a limitation in the revised manuscript as follows: “First, the sample size is smaller, and the follow-up period is shorter compared with previous studies that used public survey data to examine disability trajectories among general older populations (e.g., studies with hundreds or thousands of respondents) [61–63].” (Page 27, lines 445-448)

*6) It is unclear if the contextual factors were measured only at baseline (which is implied) or also longitudinally. Using only baseline measures is common, but it limits the ability to model how changes in social support or stigma, for example, might influence disability trajectories.*

**RESPONSE-6**: We followed the suggestion and clarified the timing of the measurement of contextual factors in the Methods section: “After participants were assigned to their respective trajectory group in each of the six disability dimensions, we conducted multinomial logistic regression models to assess the influence of intrinsic contextual factors (age, gender, education, living status, number of comorbidities, mastery) and extrinsic contextual factors (stigma, social support) measured at baseline on subsequent disability trajectories.” (Page 11, lines 212-216)

*7) Suggested Strengthened Conclusion: "The heterogeneity in disability trajectories underscores the need for personalized care. Targeting modifiable factors like stigma (e.g., through anti-stigma campaigns) and bolstering self-mastery and social support represent promising avenues for interventions designed to mitigate disability among adults aging with HIV."*

**RESPONSE-7**: Thank you. We incorporated the suggested strengthened conclusion in the Conclusion section of the main manuscript (Page 28, lines 466-470).
